# Community Involvement in the Care of Persons Affected by Podoconiosis—A Lesson for Other Skin NTDs

**DOI:** 10.3390/tropicalmed3030087

**Published:** 2018-08-16

**Authors:** Abebayehu Tora, Asrat Mengiste, Gail Davey, Maya Semrau

**Affiliations:** 1 Department of Sociology, Wolaita Sodo University, Sodo, Ethiopia; abezed@yahoo.com; 2 National Podoconiosis Action Network, Addis Ababa, Ethiopia; asrat_m@napanEthiopia.org; 3 Department of Global Health and Infection, Brighton and Sussex Medical School, Falmer Campus, University of Sussex, Brighton BN1 9PX, UK; G.Davey@bsms.ac.uk

**Keywords:** podoconiosis, lymphedema, neglected tropical diseases, NTDs, mental health, community engagement, patient involvement, stigma

## Abstract

Podoconiosis is a neglected tropical disease (NTD) characterized by lower-leg swelling (lymphedema), which is caused by long-term exposure to irritant red-clay soils found within tropical volcanic high-altitude environments with heavy rainfall. The condition places a substantial burden on affected people, their families and communities, including disability, economic consequences, social exclusion, and stigma; mental disorders and distress are also common. This paper focuses on community-based care of podoconiosis, and, in particular, the role that community involvement can have in the reduction of stigma against people affected by podoconiosis. We first draw on research conducted in Ethiopia for this, which has included community-based provision of care and treatment, education, and awareness-raising, and socioeconomic rehabilitation to reduce stigma. Since people affected by podoconiosis and other skin NTDs often suffer the double burden of mental-health illness, which is similarly stigmatized, we then point to examples from the mental-health field in low-resource community settings to suggest avenues for stigma reduction and increased patient engagement that may be relevant across a range of skin NTDs, though further research is needed on this.

## 1. Introduction

Podoconiosis is a neglected tropical disease (NTD), characterized by lower-leg swelling (lymphedema), which is found in highland areas of the tropics. It has often been confused with lymphatic filariasis, and is currently dealt with by the World Health Organization (WHO) under the lymphatic filariasis (LF) program (link: http://www.who.int/lymphatic_filariasis/epidemiology/podoconiosis/en/), but differs in that it is associated with long-term exposure to irritant tropical soils [1] rather than any parasite, virus, or bacterium. These red-clay soils are found in highland tropical areas, where ancient volcanic deposits have weathered [2] at high altitude (over 1000 m) under conditions of heavy rainfall (over 1000 mm/year). There is now strong evidence of underlying genetic susceptibility for podoconiosis [3], and endemic communities are usually aware that the condition clusters in families.

Countries with a high burden of podoconiosis include Ethiopia, Uganda, Cameroon, Kenya, and Tanzania [4]. Although rarely a direct cause of mortality, podoconiosis disables those affected through progressive leg swelling and repeated episodes of acute dermatolymphangioadenitis. These acute episodes occur frequently (reports vary from five [5] to 23 [6] episodes per year), and contribute substantially to the disability and social impact associated with podoconiosis [7,8]. Research has explored the enormous economic burden of podoconiosis on affected communities. In a southern Ethiopian zone of 1.5 million inhabitants, where the prevalence of podoconiosis is known to be 5.4%, the overall cost of podoconiosis was estimated to be in excess of US$16 million per year. In this zone, where the average income is less than US$100 per year, the direct costs to a patient were found to be US$143 per year [9]. In addition to its economic consequences, one of the most devastating characteristics of podoconiosis is stigma [10,11,12].

This article is a nonsystematic narrative review [13]. It focuses on community-based care of podoconiosis, and, in particular, the role that community involvement can have in the reduction of stigma against people affected by podoconiosis, but it also draws on other types of lymphedema, such as leprosy and LF. We start by exploring the sources and consequences of stigma among people with podoconiosis and then describe community-based approaches used to reduce stigma and provide care in Ethiopia. We then draw on examples from the field of mental health in low-resource community settings to suggest avenues for stigma reduction and increased patient engagement relevant across a range of skin NTDs. To our knowledge, this is the first review on community involvement in the care of people affected by podoconiosis, which also looks beyond NTDs to learn lessons from the mental-health field.

## 2. Sources and Consequences of Stigma in Podoconiosis

Stigma has commonly been defined as an undesirable or discrediting attribute that reduces an individual’s status in the eyes of society [14,15]. The major sources of stigma among people affected with podoconiosis include the progressive physical disability that prevents affected individuals from making a living [9,16]; the misconceptions among community members about the causes of the disease and treatment options [7,17,18,19]; fear of public identification of a disease that is known to be familial [18]; and the physical disfigurement caused as the disease advances [20].

People affected by podoconiosis, as is the case for people affected by other lower-limb lymphedemas such as leprosy and LF, are stigmatized in all areas of their daily life. Overt discrimination is common during social functions, around mate selection and marriage, when seeking employment, and in relation to leadership roles. Due to deep-seated stigmatizing attitudes within the community, affected individuals also experience self-stigma, manifested in the form of low self-esteem, suicidal ideation, and avoidance of interactions with nonaffected community members [11]. The prejudice, discrimination, and self-stigma related to podoconiosis and other lymphedemas not only compromise the psychological and social wellbeing of patients and their families, but also limit their access to healthcare and adherence with treatment. This causes a vicious circle, creating further disability, illness, and reduced economic productivity. Breaking the vicious cycle of stigma related to podoconiosis has been a top priority for community organizations and researchers to improve the quality of life of patients and their families. 

## 3. Community Interventions

Several community-based podoconiosis interventions have been implemented within Ethiopia, including provision of care and treatment, education, and socioeconomic rehabilitation. Though podoconiosis is an age-old disease, appropriate and effective means of treatment have not been understood or available until recently. Personal efforts to treat the disease using traditional knowledge have usually been counterproductive [21]. Most people affected by the disease therefore live with disfigurement and disability without receiving standardized services through the formal health system [22]. This has led to misconceptions around the possibility of cure, which, in turn, has increased the stigmatization of affected people [21]. 

### 3.1. Community-Based Care and Treatment

Over the last two decades, NGOs and faith-based organizations have played a pioneering role in providing care and treatment for people affected by the disease in vertically established outreach clinics [22,23]. In southern Ethiopia, Mossy Foot International has transformed patients who have successfully treated themselves into Community Podoconiosis Agents. Patients who have been educated to high-school completion are given full-time clinical training over a period of one week, and then become responsible for identifying patients in their own community and supporting self-care [22]. Training expert patients to provide simple, close-to-patient services is a potentially powerful solution to lack of care in countries with low formal health-services coverage. Harnessing the power of the local community to advocate for patients is likely to improve social support and diminish stigma in relation to a range of dermatologic conditions [24]. 

By halting the progress of disability and reducing the chances of illness related to acute attacks, the care that patients receive increases participation in community affairs and enhances their economic productivity, thereby improving their acceptance in society. Similar benefits have been documented for people affected by leprosy and LF, for example a leprosy self-care program in the 1990s and other leprosy and LF programs since then. Although the main function of the leprosy self-care groups was to encourage members to take responsibility for their own wound management, the group members reported a number of qualitative benefits, in particular improved self-respect and dignity and increased participation in society [25]. However, while these disease-specific examples are important, other studies suggest that integration of care and treatment into existing health systems may provide better opportunities to counter stigma than stand-alone vertical programs [26]. A systematic review conducted in 2016 suggested that joint approaches to reduce stigmatization across NTDs may be feasible given the similarities in causes, manifestations, and interventions against it [12]. Recent efforts to integrate podoconiosis care and treatment into government health structures alongside other types of lymphedema such as leprosy and LF are likely to play an important role in this regard [27].

### 3.2. Community-Based Education and Awareness

In addition to patient care and treatment, both Mossy Foot International and the International Orthodox Christian Charities (IOCC) program (in northern Ethiopia) have utilized Community Conversations or their equivalents to raise the awareness of the general population about podoconiosis causes, treatment, and prevention. These programs seek to encourage people to reflect on their place in the wider community, and to identify training, employment, and personal-development opportunities within the community sector.

The effectiveness of community-based health education run by lay people in reducing stigma has been confirmed in Ethiopia [28]. This study demonstrated that a health-education intervention that included community awareness campaigns and household skill-building activities run by lay health advisors decreased both felt and enacted stigma related to podoconiosis. In addition, awareness-raising and education amongst healthcare staff may be equally important to counter the stigmatizing attitudes and prejudices commonly held by them.

### 3.3. Community-Based Socioeconomic Rehabilitation

The final form of stigma reduction implemented in the context of podoconiosis is socioeconomic rehabilitation. This involves skills training (e.g., carpentry, masonry, hairdressing, shoemaking, etc.), enhancing entrepreneurship through revolving funds and loans, and providing educational opportunities [22]. Though the impact of socioeconomic rehabilitation in the context of podoconiosis has not been formally assessed, anecdotal evidences suggests that access to these services reduces stigma by increasing self-reliance, self-esteem, and financial independence, and acquiring new skills that widen opportunities for self-employment and access to public institutions. Participation in socioeconomic rehabilitation also influences the process of social integration and social acceptance through contributing to attitudinal change towards affected people [29].

## 4. Lessons from Community Engagement for Mental Health

Mental illness shares similar characteristics to skin NTDs in that affected persons are also heavily stigmatized and discriminated against, and are often excluded from social and economic activities [30,31,32]. Furthermore, rates of mental disorder (such as depression) and distress have been shown to be elevated amongst people with podoconiosis, LF, and other skin NTDs (which may both be an additional cause of stigma, as well as a consequence of the NTD-related stigma) [8,33,34]. Community engagement has been shown to be effective in addressing stigma, and thereby increasing uptake of and adherence to services, not only for NTDs such as podoconiosis and LF (as described above) but also for mental illness. It may therefore be helpful to look beyond NTDs, to learn lessons about community engagement from interventions used within the mental-health field, and how these might shape holistic interventions for stigmatized skin NTDs, particularly—but not only—for those who suffer the added burden of mental illness. However, further research on this is greatly needed. 

The few studies published from low- and middle-income countries (LMICs) that have aimed to increase uptake of mental-health services by reducing stigma in the local community have included awareness-raising to change community knowledge and attitudes towards people with mental illness as their core component [35,36,37]. A program implemented in several states in Nigeria [35,36], for example, led to a significant increase in the use of community-based primary-care mental-health services by training voluntary lay community health workers (CHWs) to deliver positive health messages and challenge misconceptions around mental health, alongside a media campaign of radio announcements, jingles, and newspaper coverage. The program highlighted the importance of regular ‘booster’ training (e.g., every six months) and supervision, plus possibly other initiatives (such as incentives or paid work), to sustain CHWs’ enthusiasm and the effectiveness of the program over time. Other key lessons learned from such programs have been the importance of involving and gaining buy-in from community members and leaders and health-system leaders from the start (for example, through forming of a stakeholder committee); linking raising awareness to the available services; and using ‘Training of Trainers’ approaches to achieve scale-up.

A further important finding from the mental-health field has been that interventions that include a social-contact element seem to be the most effective in reducing mental-health-related stigma and discrimination [38,39]. This can be either through direct contact with a patient, or indirect contact such as video testimonies, or affected persons sharing their experiences in newspapers, radio, TV, or posters. Whilst community-based awareness-raising interventions such as the ones outlined in the sections above may be essential in improving literacy of mental health and/or skin NTDs, it may therefore be valuable to explore further the inclusion of social-contact components within community-engagement interventions for skin NTDs and/or comorbid mental illness in order to increase their effectiveness. Contact interventions have indeed already shown successes in reducing leprosy-related stigma [40].

Service-user (patient) engagement is another area that may be as important for skin NTDs as for mental health, to empower and support affected people, and to facilitate self-care, and there are several good cases from the leprosy and LF field [27,41,42,43,44,45]. Examples of mental-health programs come from the five-year ‘Emerging mental health systems in LMICs’ (Emerald) program [46] and the seven-year ‘PRogramme for Improving Mental health carE’ (PRIME) [47], which were both implemented in countries in Africa and Asia (Ethiopia, India, Nepal, South Africa, and Uganda; plus Nigeria for Emerald). Both programs aimed to improve mental health care in these countries; whilst PRIME did so by collecting evidence on the integration and scale-up of mental health services into primary health care [47], Emerald did so by building capacity and generating evidence to enhance health-system strengthening [46]. PRIME, which also used WHO’s widely-implemented mhGAP Intervention Guide [48] within primary health care, included a range of community-based interventions within its larger program, such as awareness-raising workshops, mental-health components within Community Conversation groups, information dissemination, case detection by CHWs, community mobilization, and community-based rehabilitation [49].

Both Emerald and PRIME included the engagement of mental-health patients and their families/caregivers as an important component, and acknowledged that patient involvement is essential when developing interventions and when expanding access to integrated care services within primary-care settings in LMICs [50]. A cross-country qualitative study conducted by Emerald identified the main strategies for increasing patient involvement in health-system strengthening to be patient and caregiver mobilization and empowerment; the need for capacity-building (training) of patients/caregivers and service providers; and ensuring human rights for greater involvement [51]. Alongside this, common barriers were identified that need to be tackled and that are reminiscent of skin NTDs, such as stigma, poverty, and power differentials in the health system, including lack of knowledge of services and support in the local community [50,51,52,53]. To achieve capacity-building of mental-health patients and their families/caregivers, Emerald took a country-specific multifaceted approach that aimed to include awareness-raising, stigma reduction, information, and mobilization and engagement of patients as its key ingredients; some of the main interventions used were workshops with mental-health patients and their caregivers to raise awareness and mobilize for greater advocacy and involvement, and workshops for primary-care workers and managers to support greater patient involvement [54]. The concepts of appropriateness, reciprocity, sustainability, and equality in partnerships were identified as being important and underpinned all capacity-building activities [54,55]; these may be relevant beyond the mental-health field, including within skin NTD programs.

## 5. Looking to the Future

While several approaches to community-based delivery of care, of education and awareness, and of socioeconomic rehabilitation have been successfully used by podoconiosis (as well as leprosy and LF) programs in Ethiopia and elsewhere, there is much potential for more. Socioeconomic community-based rehabilitation in particular is an area that warrants further research.

Looking to the experience of community-based mental health services, there are still many interventions that could potentially be applied to podoconiosis and across other stigmatized skin conditions. Holistic approaches are needed that take into account not only the physical care of skin NTDs, but also integrate mental-health components and take into account the complex interplay of physical disability, mental illness, and associated stigma. At the community level, this could include awareness-raising, not only of the implications in regards to the physical health of people affected by skin NTDs, but also the mental-health implications (for example through awareness-raising workshops, the inclusion of mental-health components within Community Conversation groups, or information dissemination). Social-contact elements may be particularly useful in this respect. Similarly, patient engagement, for example through patient groups, is vital in order to empower and support affected people, and to facilitate self-care. Services should be integrated so that patients receive care not only for their physical disabilities but also any mental distress or disorder; this can be achieved by making integrated services readily available in nearby primary healthcare facilities and/or in the community, for instance by training (and then providing regular supervision for) primary healthcare staff and/or community health workers in case detection and/or treatment of mental disorders, as well as physical healthcare. Stigma-reduction activities within this training may be important to counter any negative attitudes or prejudices. Further research is required to adapt and evaluate these programs in a range of disease and country contexts.
